# Early Time-Restricted Eating, Circadian Alignment, and Cognitive Preservation in Older Adults: A Neurobiological Hypothesis

**DOI:** 10.3390/neurosci7050106

**Published:** 2026-09-30

**Authors:** Bianca Farias Lima, Gioconda Ferreira, Fernanda de Oliveira Calvente, Yasmin da Silva Costa, Mariana Alencar, Jhenifer de Paiva Quadros, Hércules Rezende Freitas

**Affiliations:** 1 IBMR School Clinic, Escola de Ciências da Saúde, Centro Universitário IBMR, Barra da Tijuca, Rio de Janeiro 22631-002, RJ, Brazil; byafariaslima@gmail.com (B.F.L.); giocondampa@gmail.com (G.F.); fernandacalvente@gmail.com (F.d.O.C.); yasmindasilvacosta@rocketmail.com (Y.d.S.C.); marianaproenca207@gmail.com (M.A.); jheniferdpq@gmail.com (J.d.P.Q.); 2 Health Informatics Laboratory (LabInfoS), Departamento de Ciências Médicas Integradas, Faculdade de Ciências Médicas, Centro Biomédico, Universidade do Estado do Rio de Janeiro, Cabo Frio 28905-320, RJ, Brazil

**Keywords:** chrononutrition, circadian rhythms, cognitive decline, time-restricted eating, glymphatic system, ageing

## Abstract

Nearly half of dementia cases are attributed to fourteen modifiable risk factors listed by the 2024 Lancet Commission; meal timing is not among them. Disrupted daily (circadian) rhythms are linked to faster neurodegeneration, yet it is unknown whether meal timing affects cognitive ageing. We review the evidence for the hypothesis that early time-restricted eating (eTRE) helps preserve cognition in older adults. We define eTRE as eating within a window of no more than 10 h, with at least 80% of daily energy eaten before 16:00. We evaluate three candidate pathways: daily rhythms in the removal of amyloid-beta and tau from the brain (glymphatic clearance), daily rhythms that keep brain inflammation low, and clock genes that support memory-related plasticity in the hippocampus. Nutrient-sensing and gut microbiota pathways are considered as alternatives. An AI co-scientist analysis, checked by the authors, suggested a “Dual-Gating” model: finishing meals early may lower nervous-system arousal and insulin levels at night, and both changes may favour brain clearance during sleep. We outline a three-arm trial (eTRE, late time-restricted eating, habitual eating) that tests whether any cognitive benefit is explained by changes in markers of clearance and inflammation. Meal timing remains a candidate exposure that needs prospective testing.

## 1. Introduction

By 2050, an estimated 139 million people will live with dementia [1]. The 2024 Lancet Commission identified fourteen modifiable risk factors that together account for about 45% of dementia cases across the life course [2]: less education, hearing loss, high low-density lipoprotein (LDL) cholesterol, depression, traumatic brain injury, physical inactivity, diabetes, smoking, hypertension, obesity, excessive alcohol consumption, social isolation, air pollution, and untreated vision loss [2]. Meal timing is not on this list. Chrononutrition studies how the timing of food intake interacts with the body’s internal clocks [3]. It offers a plausible route by which eating schedules could affect brain health, separately from what or how much people eat.

The human circadian system runs on cycles of roughly 24 h. A central pacemaker in the suprachiasmatic nucleus (SCN) coordinates peripheral clocks in organs such as the liver, kidney, and pancreas. Feeding time is a potent non-photic time cue (zeitgeber) for these peripheral clocks: in humans, this principle underlies chrononutrition [3,4], and in mice, restricted feeding uncouples them from the SCN [5]. Circadian rhythms weaken with age: hormonal rhythms lose amplitude, sleep and wakefulness become fragmented, and central and peripheral clocks drift apart [6]. These changes appear early in the course of neurodegenerative disease. Some authors therefore propose that circadian dysfunction may contribute to the disease process and is not only a consequence of it [7,8].

The idea that feeding schedules affect health is not new. In mice, time-restricted feeding (TRF) prevented metabolic disease without reducing caloric intake [9]. In patients with Alzheimer’s disease (AD), a four-month TRF intervention was associated with better cognition through a gut microbiota pathway [10]. The present review builds on this work in three ways: First, it asks whether the circadian position of the eating window, and not only its length, matters for cognitive ageing. Second, it proposes a specific and testable mechanistic model that links meal timing to nocturnal brain clearance. Third, it describes a trial design that can separate timing from fasting duration.

Throughout the text, we use three evidence levels: Established evidence refers to findings replicated in humans or in multiple independent experimental studies. Biological plausibility refers to links supported mainly by preclinical or indirect human data. Hypothesis-specific predictions are propositions that have not yet been tested and that the proposed trial is designed to test (Table 1). Following common usage, we use “time-restricted eating” (TRE) for human studies and “time-restricted feeding” (TRF) for animal studies. We keep “TRF” only where the original human study used that term.

## 2. Hypothesis Development and Use of Generative AI

### 2.1. Human-Authored Starting Point

The hypothesis was first written by the student authors (B.F.L., G.F., F.O.C., Y.S.C., M.A.) as an undergraduate nutrition thesis under the supervision of J.P.Q. and H.R.F. That draft contained the operational definition of aligned eating, the three mechanistic pathways (glymphatic, neuroimmune, synaptic), the directed acyclic graph, the principal outcomes (cognitive batteries, plasma p-tau, and the DTI-ALPS index), and a two-arm randomized design. Narrative literature searches were carried out in PubMed and Google Scholar using combinations of the terms “chrononutrition”, “time-restricted eating”, “time-restricted feeding”, “meal timing”, “circadian”, “glymphatic”, “cognition”, “dementia”, and “older adults”. Priority was given to randomized trials, longitudinal cohorts, and mechanistic studies.

### 2.2. AI Co-Scientist Analysis

On 17 June 2026, we used Google Hypothesis Generation (AI co-scientist, Google Labs), a multi-agent system that generates, critiques, ranks, and refines research hypotheses through a tournament-style process [28]. The exact prompt written by the authors, based on the thesis draft, was recorded by the system as the “Research Summary” of the run specification. At the goal-specification stage, the system structured this prompt into four focus areas and eight preferences, which the authors accepted before the run started. The complete run specification is reproduced verbatim in Supplementary File S1. It contains the Research Summary (the prompt), the focus areas, the preferences, and the data sources enabled (PubMed, arXiv, and bioRxiv, together with the UniProt, Open Targets, and ChEMBL tools). The prompt itself already required that any cognitive benefit be at least partly mediated by glymphatic or inflammatory change, and it specified the target population, the eTRE definition, and the primary outcomes. The three-arm comparison (eTRE 08:00–16:00 versus late TRE 12:00–20:00 versus habitual eating) does not appear in the prompt; it was introduced among the preferences at the goal-specification stage (the thesis draft had proposed a two-arm trial). The system then produced a knowledge base, a summary report, and several dozen ranked idea reports (all available on request).

### 2.3. What the AI Contributed and How It Was Verified

The system contributed two main elements: the three-arm design described above, and the “Dual-Gating” model (Section 3.3), which appeared in several of the top-ranked research directions. We also adopted three methodological refinements from the output: a feasibility pilot before the main trial, standardized timing of biological sampling, and the explicit use of structural equation modelling (SEM) for mediation testing. We did not adopt several other system-generated mechanisms (for example, a proposed insulin–matrix metalloproteinase-9–dystrophin complex axis and a ketone body–HDAC3 epigenetic axis), because we could not trace each of their steps to primary evidence. For every element that we retained, the authors took the following steps: (i) Each mechanistic step was checked against primary publications identified independently of the system. (ii) Every reference in this manuscript was checked manually against the publisher’s record or PubMed. (iii) Links lacking direct evidence were labelled here as hypothesized (Table 1). The scientific case made in this review rests on the cited literature and on the authors’ reasoning, not on the AI output. We report the AI contribution so that readers can judge where each idea came from.

## 3. The Chrononutrition Hypothesis

We hypothesize that eTRE protects against age-related cognitive decline through circadian and neurobiological mechanisms. We further hypothesize that this effect is at least partly separable from the effects of caloric intake, diet composition, and weight loss. Sleep may carry part of the effect (Section 4.2); we do not assume that the effect is independent of sleep.

### 3.1. Operational Definition

For testability, eTRE is defined by three concurrent criteria: (a) at least 80% of daily energy intake consumed before 16:00; (b) a total daily eating window of no more than 10 h, starting after habitual wake time; and (c) at least 3 h between the last intake and habitual sleep onset. With a 16:00 cut-off, criterion (c) is usually exceeded by a wide margin (about 6–7 h for a bedtime of 22:00–23:00). The 80% threshold gives observational studies a tolerance for small late snacks. In the trial, the eTRE arm is prescribed a fixed 08:00–16:00 window (Section 5), so the two definitions agree. An earlier draft used a 19:00 cut-off. We abandoned it because a pattern in which up to 20% of energy is eaten after 19:00 is hard to reconcile with a long pre-sleep fast, and because it does not clearly separate early from late eaters. Eating patterns can be measured with time-stamped digital food diaries, wrist actigraphy, and a validated chronotype instrument, the Morningness–Eveningness Questionnaire (MEQ).

This pattern differs from late time-restricted eating (lTRE; for example, 12:00–20:00); lTRE has the same fasting duration but places the metabolic load later in the circadian day, when glucose tolerance is lower [12]. The hypothesis is therefore about when food is eaten, not what or how much.

### 3.2. Mechanistic Foundation

**Figure 1:**
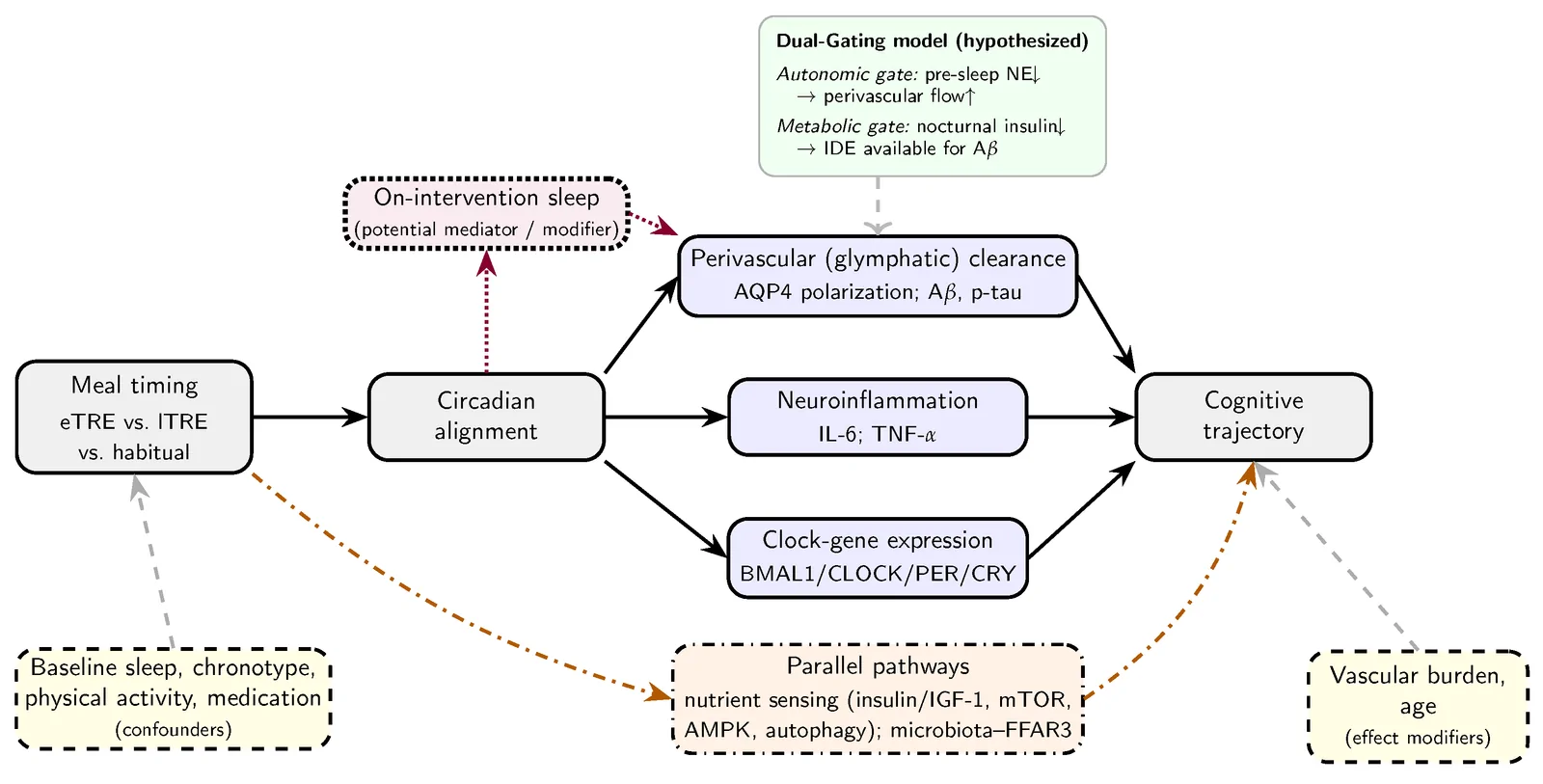
Directed acyclic graph (DAG) of the hypothesized pathways. Solid arrows are hypothesized causal links whose evidence status is given in Table 1; none has yet been established for meal timing in humans. The dotted path indicates that sleep during the intervention may lie on the causal path (mediator) or modify its effect. Baseline sleep, chronotype, physical activity, and medication are pre-randomization confounders, balanced by randomization and adjusted for. Dash-dotted arrows are parallel or competing pathways (nutrient sensing; gut microbiota). Vascular burden and age may modify the clearance pathway. The Dual-Gating model (inset), proposed through the AI co-scientist analysis [28] and verified against the primary literature by the authors, specifies two hypothesized mechanisms that could distinguish eTRE from lTRE despite equal fasting duration. In the inset, upward arrows (↑) indicate an increase and downward arrows (↓) a decrease. Abbreviations: eTRE/lTRE, early/late time-restricted eating; AQP4, aquaporin-4; Aβ, amyloid-beta; p-tau, phosphorylated tau; IL-6, interleukin-6; TNF-α, tumour necrosis factor-alpha; IGF-1, insulin-like growth factor-1; mTOR, mechanistic target of rapamycin; AMPK, AMP-activated protein kinase; FFAR3, free fatty acid receptor 3; NE, norepinephrine; IDE, insulin-degrading enzyme.

Late or irregular eating can shift peripheral clocks out of phase with the SCN [5]. This disrupts the rhythmic expression of the core clock genes (BMAL1, CLOCK, PER, and CRY) in several tissues. We propose three downstream pathways through which this disruption could harm cognition, each of which could be reversed by eTRE (Figure 1). The evidence status of each link is summarized in Table 1.

**Perivascular (glymphatic) Clearance (primary pathway):** The glymphatic model describes cerebrospinal fluid (CSF) entering the brain along periarterial spaces, exchanging with interstitial fluid (ISF), and leaving along perivenous routes [29]. The water channel aquaporin-4 (AQP4) is concentrated (“polarized”) at astrocytic endfeet that surround blood vessels, and this polarization supports efficient exchange [29]. In mice, the interstitial space expands during sleep, and clearance of amyloid-beta (Aβ) increases [15]. Clearance also follows an intrinsic circadian rhythm that depends on AQP4 [16], and glucocorticoid rhythms help coordinate it with the sleep–wake cycle [30]. Arterial pulsations drive perivascular CSF flow [31]. In non-rapid eye movement (NREM) sleep, slow oscillations of norepinephrine (NE) produce rhythmic vasomotion that propels CSF [17]. In humans, NREM sleep is accompanied by large, coupled oscillations in neural activity, blood volume, and CSF flow [18].

Each of these drivers changes with age. Aged mice show loss of AQP4 polarization and markedly reduced perivascular exchange [32]. In human brains, loss of perivascular AQP4 localization is associated with AD pathology and cognitive status [33]. In mice, acutely raising blood pressure alters the pulsation of the arterial wall and increases backflow, which reduces net perivascular CSF flow [31]; whether the arterial stiffening of ageing acts in the same way has not been tested. Cerebral small-vessel disease and enlarged perivascular spaces become common in later life and may themselves impair clearance [34]. In a population aged ≥65 years, these structural factors could limit or modify any benefit of meal timing, and they must be measured (Section 5). The model is also debated. One mouse study found reduced, rather than increased, brain clearance during sleep and anaesthesia [19]. Most direct evidence comes from rodents. Circadian disruption is associated with faster Aβ and tau pathology in experimental models [20]. In older adults, fragmented rest–activity rhythms are associated with preclinical AD pathology [8]. Whether meal timing changes glymphatic function in humans has not been tested.

**Neuroimmune Rhythmicity (secondary pathway):** Microglia have their own circadian clocks, and circadian disruption promotes neuroinflammation and weakens microglial handling of Aβ [20]. In mice, loss of BMAL1 causes astrocyte activation and synaptic damage [35]. We predict that late eating raises nocturnal interleukin-6 (IL-6) and tumour necrosis factor-alpha (TNF-α) in humans, and that eTRE restores their daily rhythm. This remains a hypothesis-specific prediction.

**Synaptic Plasticity (tertiary pathway):** Hippocampal memory processes, including cAMP–CREB signalling, vary across the day and depend on clock genes [36]. Disrupting the core clock impairs hippocampus-dependent memory in mice [37]. Whether eTRE preserves these processes in older humans is not known.

### 3.3. The Dual-Gating Model (Hypothesized)

The AI co-scientist analysis (Section 2) proposed a “Dual-Gating” model to explain why eTRE might outperform lTRE even when fasting duration is the same. We present the model as a set of hypothesized links, each with its supporting evidence and its main gap (Table 1).

1. **Autonomic Gate (hypothesized):** Food intake acutely increases sympathetic nerve activity in humans [22]. In mice, NE levels regulate interstitial space volume [15], and slow NE oscillations during NREM sleep drive glymphatic flow [17]. We hypothesize that finishing the last meal several hours before sleep (about 6 h with eTRE versus about 2–3 h with lTRE) lowers sympathetic tone at sleep onset. This would allow normal NE dynamics and perivascular flow during early slow-wave sleep. Direct evidence that meal timing changes brain NE dynamics or perivascular flow in humans is lacking.
2. **Metabolic Gate (hypothesized):** Insulin-degrading enzyme (IDE) degrades both insulin and Aβ, and insulin competes with Aβ for this enzyme [23]. IDE deficiency raises brain Aβ levels in mice [24]. In older adults, raising insulin levels increases CSF Aβ42 [25]. Glucose tolerance is lower later in the day, so identical meals eaten late produce higher glucose and insulin responses [12]. Under controlled conditions, late eating also increases hunger, lowers energy expenditure, and alters adipose-tissue gene expression [13]. Upstream, amino acid and nutrient signals act through mTORC1 to control insulin production by pancreatic beta-cells [38,39]. Timing of the nutrient load may therefore shape nocturnal insulin exposure. We hypothesize that lower insulin at night leaves more IDE capacity for Aβ breakdown. Whether this occurs in the human brain at physiological insulin levels is unknown.

Together, the two gates offer a falsifiable explanation of why the circadian position of the eating window might matter beyond its length. The three-arm trial in Section 5 tests this directly.

### 3.4. Alternative and Parallel Pathways

The glymphatic pathway is not the only way in which eTRE could affect the ageing brain. Fasting and meal timing act on conserved nutrient-sensing pathways: insulin/insulin-like growth factor-1 (IGF-1) signalling, the mechanistic target of rapamycin (mTOR), and AMP-activated protein kinase (AMPK) [40,41,42]. These pathways control autophagy, proteostasis, and mitochondrial function, and they underlie many of the effects of dietary restriction on healthspan [43,44]. In flies, the benefits of intermittent TRF on lifespan require circadian regulation of autophagy [45]. In humans, early TRE changed the expression of clock and autophagy genes [11]. These pathways may act alongside the glymphatic pathway or explain the same benefits without it.

A second alternative is the gut. Feeding schedules reshape the daily rhythms of the gut microbiome [46], and microbial metabolites signal to the brain [47]. In a study that included patients with AD, the cognitive benefit of TRE was attributed to a *Bifidobacterium pseudolongum*–propionic acid–free fatty acid receptor 3 (FFAR3) pathway [10]. We therefore treat nutrient sensing and the microbiota as competing explanations, to be measured and tested in the trial (Section 5). If cognition improves without glymphatic change, the result would favour these alternatives.

## 4. Supporting Evidence and Evaluation

The strongest mechanistic support comes from Hablitz et al. [16]. In mice, they showed that glymphatic clearance follows an intrinsic circadian rhythm, peaks during the rest phase, and loses this rhythm when AQP4 is deleted. Madamanchi et al. [20] reviewed evidence that circadian disruption promotes oxidative stress, neuroinflammation, and glymphatic dysfunction and accelerates amyloid and tau pathology in experimental models. In mice fed a high-fat diet, TRF prevented metabolic disease without reducing caloric intake [9,48]. In humans, early TRE improved insulin sensitivity, blood pressure, and oxidative stress without weight loss under controlled feeding [14]. In a randomized trial in adults without obesity, early TRE improved insulin sensitivity more than mid-day TRE [49].

Zhao et al. [10] reported that four months of time-restricted eating (termed TRF by the authors) improved cognition in patients with AD. In mouse models, they attributed this benefit to a gut microbiota–propionic acid–FFAR3 pathway rather than to glymphatic clearance. This finding matters for our hypothesis. It shows that restricting meal timing can influence cognition in humans. It also shows that the glymphatic mechanism has not yet been tested in a randomized trial.

In 2945 community-dwelling older adults from a British cohort, assessed repeatedly between 1983 and 2017, Dashti et al. [50] found that later breakfast timing was associated with multimorbidity and with higher mortality. This supports the relevance of meal timing to health in later life, although the study did not examine cognitive outcomes. In a cross-sectional study of 883 adults in Sicily, an eating window shorter than 10 h was associated with lower odds of cognitive impairment, as was eating breakfast, whereas eating dinner was not [26]. A scoping review of 12 studies [27] found that time-restricted feeding, intermittent fasting, and eating earlier in the day were mostly associated with better cognition. Most of the included studies were cross-sectional with small samples, limiting causal inference. Overall (Table 2), the evidence supports biological plausibility and justifies a formal test. It does not establish that eTRE protects cognition.

### 4.1. Limitations and Apparently Contrary Evidence

The human evidence is mostly observational and heterogeneous. Samples are small, follow-up is short, and cognition is rarely the primary endpoint. The cross-sectional association reported by Currenti et al. [26] between a shorter eating window and lower odds of cognitive impairment is consistent with the hypothesis, but it cannot exclude reverse causality or confounding. It also does not separate the length of the eating window from its timing, although the association with eating breakfast, but not dinner, is compatible with a role for timing. The evidence is also not uniformly favourable: the scoping review by Ding et al. identified one study in which TRF was associated with a higher prevalence of cognitive impairment in older adults [27].

There are also important translational gaps. First, the evidence for circadian and sleep regulation of glymphatic clearance comes almost entirely from rodents, which are nocturnal and have different sleep architecture and brain geometry. Second, the rodent findings are not fully consistent [19]. Third, human glymphatic function cannot yet be measured non-invasively and directly. Fourth, no human study has tested whether meal timing changes brain clearance, brain NE dynamics, or central IDE activity. Fifth, human chrononutrition trials have mostly enrolled middle-aged adults with metabolic endpoints, over weeks to months [51,52]. Sixth, age-related vascular disease [34] could weaken any benefit in the target population.

### 4.2. Sleep: Confounder, Mediator, or Effect Modifier?

Meal timing and sleep are hard to disentangle. Circadian misalignment and poor sleep share mechanisms, such as effects on clearance, melatonin, and cortisol, and they often occur together in older adults. Their causal relationship depends on when sleep is measured. Sleep quality before randomization is a confounder in observational studies and a baseline covariate in a trial. Sleep during the intervention may be changed by eTRE: eating close to bedtime is associated with poorer sleep [53], and a longer pre-sleep fast could alter sleep timing, slow-wave sleep, autonomic tone, or melatonin and cortisol rhythms. On-intervention sleep is therefore a potential mediator, particularly for the autonomic gate. Sleep could also modify the effect, because clearance benefits may require adequate slow-wave sleep. Adjusting for on-intervention sleep as if it were a confounder would remove part of the effect that we aim to estimate. We therefore revised the causal model (Figure 1). The trial will treat baseline sleep as a covariate, treat on-intervention sleep as a candidate mediator in the mediation analysis, and test sleep as an effect modifier in pre-specified interaction analyses.

### 4.3. Reverse Causality and Sarcopenia Risk

Reverse causality is a further concern in observational data. Early cognitive impairment affects executive function and memory before diagnosis and can make meal times less regular [2]. This would create an association with the causal direction reversed. Randomized designs avoid this problem because the intervention is assigned before outcomes are measured.

Finally, compressing the eating window in older adults could increase the risk of sarcopenia. Older adults need more protein (1.0–1.2 g/kg/day for healthy older adults and 1.2–1.5 g/kg/day for those with acute or chronic disease) [54,55]. Spreading protein evenly across meals increased 24-hour muscle protein synthesis in healthy adults of middle age [56], and guidelines for older adults recommend the same distribution [54,55]. In one TRE trial, participants lost a substantial share of their weight as lean mass [57]. Section 5.2 describes how the trial would monitor and prevent this risk.

## 5. A Critical Experimental Test

### 5.1. Design and Intervention Arms

The critical experiment is a three-arm randomized controlled trial (RCT). The arms are as follows: (1) eTRE, with all intake between 08:00 and 16:00; (2) lTRE, with all intake between 12:00 and 20:00, which gives the same fasting duration (≈16 h) but places the metabolic load closer to sleep onset; and (3) habitual eating (control; baseline window ≥13 h). Allocation will use computer-generated sequences with concealment, stratified by chronotype (MEQ) and by cognitive status. Cognitive assessors and imaging and laboratory analysts will be blinded to allocation. The eTRE-versus-lTRE comparison, with equal fasting duration but different circadian timing, is the core of the design. It tests whether timing, rather than restriction itself, drives the hypothesized effects. Adherence will be defined as ≥80% of days with all logged intake inside the assigned window (±30 min). It will be monitored through time-stamped photographic food logs and, in a subsample, continuous glucose monitoring.

### 5.2. Participants and Safety Monitoring

Eligible participants will be adults aged ≥65 years without dementia but with increased cognitive risk. Increased risk is defined as mild cognitive impairment or a first-degree family history of AD, together with a baseline eating window of ≥13 h. Exclusion criteria will include frailty (Fried phenotype [58]), probable sarcopenia or confirmed sarcopenia (EWGSOP2 criteria [59]), body mass index < 22 kg/m^2^ or unintentional weight loss > 5% in the preceding 6 months, insulin or sulfonylurea therapy (hypoglycaemia risk), night-shift work, and advanced sleep-phase disorder.

Each participant will receive an individualized protein target of 1.0–1.2 g/kg/day. The target will be 1.2–1.5 g/kg/day for those with chronic disease and for those with low muscle mass at baseline [54,55]. Protein intake will be spread across the meals inside the window, with about 25–30 g per main meal [54,55,56]. All arms will receive the same guidance on resistance and aerobic activity [55]. Lean mass will be measured by dual-energy X-ray absorptiometry (DXA) at baseline, 6 months, and 12 months. Handgrip strength and the Short Physical Performance Battery (SPPB) will be measured every 3 months. Participants will be withdrawn from the assigned window, and followed up, if they lose more than 5% of body weight, if handgrip strength or SPPB score falls below sarcopenia cut-offs [59], or if they have recurrent symptomatic hypoglycaemia.

### 5.3. Control of Diet, Weight, and Co-Interventions

Meal timing can only be credited with an effect if the other dietary factors are held constant across arms. To achieve this, all arms will receive energy prescriptions intended to maintain body weight, based on estimated requirements. They will also receive the same dietary-quality guidance and meal templates with matched macronutrient composition. Body weight will be checked every 2 weeks, and energy advice will be adjusted to keep weight within ±2% of baseline. Residual changes in weight, HbA1c, and HOMA-IR will be modelled as competing mediators. Diet quality will be scored from repeated 24 h recalls. Medication changes will be recorded at every visit. Physical activity and sleep duration will be monitored by wrist actigraphy (≥7 consecutive days at each assessment). All biological samples will be collected at a fixed clock time after a standardized overnight fast. This will avoid confounding by differences in time since the last meal.

### 5.4. Outcomes and Assessment Schedule

Primary cognitive outcomes are episodic memory and executive function. These will be measured with the Preclinical Alzheimer Cognitive Composite-5 (PACC-5) [60]. PACC-5 combines word-list and story recall, digit-symbol coding, and semantic fluency, and it is more sensitive to early decline than screening tests. The Montreal Cognitive Assessment (MoCA) will be a secondary outcome. The primary biological outcome is the trajectory of plasma p-tau217, measured with an ultra-sensitive assay; p-tau217 rises early in the course of AD [61]. Plasma p-tau181 is a secondary biological outcome. The DTI-ALPS index is a mechanistic outcome (Section 5.5). Further secondary biomarkers are serum neurofilament light chain (NfL), the amplitude of the salivary cortisol awakening response (a marker of circadian robustness), diurnal IL-6 and TNF-α (neuroimmune rhythmicity), fasting insulin and glucose, and faecal short-chain fatty acids (microbiota pathway). Table 3 gives the assessment schedule. The mediator (DTI-ALPS and biomarker change at 6 months) will be measured before the primary cognitive endpoint (12 months), so that the mediation analysis respects temporal order [62].

### 5.5. Interpreting DTI-ALPS

DTI-ALPS compares water diffusivity along the direction of medullary veins with diffusivity along the neighbouring projection and association fibres [63]. It is an MRI surrogate that is associated with perivascular and glymphatic function. It does not measure glymphatic flux. The index is taken from a small region of deep white matter, where perivascular spaces make up a small fraction of the tissue. It is also affected by white-matter microstructure, fibre geometry, vascular pathology, age, and acquisition and processing choices [64,65]. For these reasons, DTI-ALPS will be analysed together with free-water and white-matter integrity metrics. Perivascular-space burden and white-matter hyperintensities will be included as covariates. Imaging protocols will be harmonized across sites. Conclusions about clearance will require agreement between DTI-ALPS and fluid biomarkers (the p-tau217 trajectory and NfL). Where feasible, a subsample could undergo more direct assessment, such as dynamic CSF-flow MRI during sleep [18]. Invasive tracer methods are unlikely to be acceptable in a prevention trial.

### 5.6. Trial Duration

The minimum duration is 12 months, for three reasons: First, cognitive change in at-risk older adults is slow; multidomain prevention trials such as FINGER used two years to detect effects on cognition [66]. Second, plasma p-tau217 changes over years rather than months [61], so shorter trials would mainly detect metabolic effects. Third, the design needs an intermediate time point (6 months) for the mediators. To limit drop-out over this period, the main trial will be preceded by a 3-month feasibility pilot (n=20–30). The pilot will estimate adherence, retention, and variance parameters. Retention will be supported by regular dietitian contact, flexible visit scheduling, and transport support. Missing data will be handled by multiple imputation under a missing-at-random assumption, with sensitivity analyses for departures from it.

### 5.7. Sample Size and Analysis

The primary endpoint for sample size is the 12-month change in PACC-5 in the eTRE-versus-lTRE comparison. It will be analysed by analysis of covariance adjusted for baseline. We assumed a standardized between-group difference of d=0.3, a two-sided α=0.05, 80% power, and a baseline-to-12-month correlation of ρ=0.7 (to be confirmed in the pilot). The formula $n=2{\left(z_{1-\alpha /2}+z_{1-\beta }\right)}^{2}\left(1-\rho^{2}\right)/d^{2}$ then gives about 90 evaluable participants per arm. Allowing for 25% attrition, 120 participants per arm (360 in total) should be randomized; with 30% attrition, about 130 per arm are needed. The eTRE-versus-habitual comparison will be tested only after the primary comparison is significant (fixed-sequence procedure), so no further α adjustment is needed.

The mediation analysis is secondary and was powered separately. Its power depends on the product of the path from intervention to mediator (*a*) and the path from mediator to outcome (*b*) [67]. We ran a Monte Carlo simulation (2000 replicates; joint-significance test; 90 evaluable participants per arm; habitual-eating arm included as a covariate group). It showed ≥80% power when a≥0.45 SD and b≥0.25. Power was 76% for a=0.40 and b=0.30, and only about 50% for a=0.30. If the pilot suggests a≈0.35, about 140–150 evaluable participants per arm would be needed. The final sample size will therefore be the larger of the primary-endpoint and mediation estimates, recalculated by simulation from the pilot parameters [68]. Mediation will be estimated with SEM using bias-corrected bootstrap confidence intervals. The mediators will be DTI-ALPS change, p-tau217 trajectory, and inflammatory change at 6 months. On-intervention sleep and metabolic markers will be included as parallel mediators, and baseline covariates will be adjusted for (sleep quality, chronotype, physical activity, protein intake, and medication use). The analysis will also estimate interventional (in)direct effects to account for exposure–mediator interaction [69]. Because the mediators are not randomized, mediation results will be interpreted as supportive rather than conclusive evidence of mechanism. If cognition improves with eTRE without measurable glymphatic or inflammatory change, the Dual-Gating model is weakened. Such a result would point instead to the alternative pathways in Section 3.4, including the gut microbiota pathway reported by Zhao et al. [10].

Table 4 assesses the hypothesis against standard criteria for scientific hypotheses [70,71].

## 6. Implications and Future Directions

If the hypothesis is confirmed, advice on when to eat could eventually be added to prevention programmes without changing diet composition, caloric intake, or medication. Few recognized risk factors share this property. Any such advice must first be shown to be safe for muscle mass and nutritional status in older adults.

The Lancet Commission has added risk factors over time. Its decisions have relied on convergent observational evidence, plausible mechanisms, and estimates of population-attributable fraction, not only on randomized trials [2]. Physical inactivity and social isolation were among the risk factors in the 2017 report; excessive alcohol, traumatic brain injury, and air pollution were added in 2020. Meal timing does not yet meet these standards. There are no consistent prospective data linking meal timing to cognitive outcomes, and no estimate of attributable fraction. Meal timing should therefore be regarded as a candidate behavioural exposure that requires prospective validation. Trials such as the one proposed here, together with cohort analyses of habitual meal timing, would provide the evidence needed to judge whether meal timing should eventually be considered as a modifiable risk factor.

At a theoretical level, the hypothesis treats circadian decline as a possible contributor to neurodegeneration, not only a consequence of it. Sleep and physical activity are thought to protect the brain partly through the same mechanisms discussed here. If eTRE acts on these mechanisms by a different route—namely, the timing of meals—dementia prevention research would gain a new intervention to test. Such an intervention would be cheap, easy to adopt, and could be offered to large populations.

This work also illustrates one way to use AI in hypothesis development. We documented the input (Supplementary File S1), specified which elements the system contributed, and checked each retained element against the literature (Section 2). In our experience, the system helped turn a general hypothesis into a more specific and falsifiable proposal. However, the AI output was a source of candidate ideas, not of evidence, and several of its proposals were rejected on review.

The hypothesis may not apply to some populations. In advanced dementia, clock circuits are already severely disrupted. In chronic night-shift workers, social time cues are inverted. In people with advanced sleep-phase disorder, the optimal eating window may differ. In long-term care facilities, meal times are set by the institution rather than by residents. Individual eTRE protocols would therefore be hard to deliver without the facility’s cooperation. On the other hand, meal schedules differ between facilities. Comparing residents of facilities with earlier and later meal times could provide observational evidence to complement controlled trials.

## 7. Conclusions

Meal timing is a biologically plausible but untested candidate modifiable exposure for cognitive ageing. Three candidate pathways (glymphatic clearance, neuroimmune rhythmicity, and synaptic plasticity) could link early time-restricted eating to brain health. Nutrient-sensing and gut microbiota pathways are credible alternatives. An author-verified AI co-scientist analysis contributed a hypothesized Dual-Gating mechanism whose individual steps can each be tested. We propose a three-arm randomized trial (eTRE, lTRE, and habitual eating) with safety monitoring and a pre-specified mediation analysis as the critical test. A positive result would justify larger prospective studies of meal timing in dementia prevention.

## Figures and Tables

**Table 1:** Evidence status of each link in the proposed causal chain.

Link	Status	Main Supporting Evidence	Main Gap
Meal timing entrains peripheral clocks	Established (animal); supported (human)	Restricted feeding uncouples peripheral clocks from the SCN [5]; early TRE alters clock-gene expression in humans [11]	Brain clock effects in humans unknown
Late eating worsens metabolic regulation	Established (human)	Lower evening glucose tolerance [12]; late eating raises hunger and lowers energy expenditure [13]; early TRE improves insulin sensitivity [14]	Few data in adults ≥ 65 years
Circadian rhythm and sleep regulate perivascular clearance	Established (rodent); indirect (human)	[15,16,17,18]; contested by [19]	Direct human flux measures are invasive or indirect
Circadian disruption accelerates Aβ/tau pathology	Supported (animal); associative (human)	[8,20]; sleep–wake cycle regulates interstitial Aβ in mice [21]	Causality in humans
Autonomic gate: early last meal → lower pre-sleep NE → higher perivascular flow	Hypothesized	Food raises sympathetic activity [22]; NE regulates clearance [15,17]	No study links meal timing to brain NE or flow
Metabolic gate: lower nocturnal insulin → more IDE capacity for Aβ	Hypothesized	IDE substrate competition [23,24]; insulin raises CSF Aβ [25]	Not shown at physiological insulin levels in the brain
eTRE improves cognition in older adults	Hypothesis-specific prediction	TRE/TRF with cognitive benefit in AD, via microbiota [10]; observational associations [26,27]	No RCT comparing early with late TRE on cognition
Benefit mediated by clearance/inflammation	Hypothesis-specific prediction	None	Tested by pre-specified mediation analysis

SCN = suprachiasmatic nucleus; TRE = time-restricted eating; TRF = time-restricted feeding; NE = norepinephrine; IDE = insulin-degrading enzyme; CSF = cerebrospinal fluid; AD = Alzheimer’s disease; RCT = randomized controlled trial.

**Table 2:** Key supporting evidence and identified gaps.

Study	Domain	Key Finding/Relevance
Hablitz et al., 2020 [16]	Glymphatic biology (mouse)	Intrinsic AQP4-dependent circadian rhythm of glymphatic clearance with a rest-phase peak; lost after AQP4 deletion.
Hauglund et al., 2025 [17]	Glymphatic biology (mouse)	NE-driven slow vasomotion propels CSF during NREM sleep; suppressed by zolpidem.
Kress et al., 2014 [32]	Ageing (mouse)	Ageing reduces perivascular exchange and AQP4 polarization.
Madamanchi et al., 2025 [20]	AD pathology (review)	Circadian disruption linked to faster Aβ and tau pathology via oxidative stress, neuroinflammation, and glymphatic dysfunction.
Hatori et al., 2012 [9]	Chrononutrition (mouse)	TRF prevents metabolic disease in mice on a high-fat diet without reducing caloric intake.
Sutton et al., 2018 [14]	Early TRE (human)	Early TRE improved insulin sensitivity and blood pressure without weight loss under controlled feeding.
Zhao et al., 2025 [10]	TRE and cognition (human and mouse)	Four months of TRE improved cognition in patients with AD; mechanism attributed to gut microbiota–propionic acid–FFAR3, distinct from and complementary to the glymphatic pathway.
Dashti et al., 2025 [50]	Meal-timing epidemiology	Later breakfast associated with multimorbidity and mortality in 2945 British older adults; indirect evidence, non-cognitive endpoints.
Ding et al., 2025 [27]	Meal timing and cognition	Scoping review of 12 studies: TRF, intermittent fasting, and earlier meals mostly associated with better cognition; one study reported a higher prevalence of cognitive impairment with TRF; mostly cross-sectional designs with small samples.
Currenti et al., 2021 [26]	TRE and cognition (cross-sectional)	In 883 adults in Sicily, an eating window <10 h (OR 0.28) and eating breakfast, but not dinner, were associated with lower odds of cognitive impairment; duration and timing of the window not separated.
Livingston et al., 2024 [2]	Policy context	Fourteen modifiable risk factors explain ∼45% of dementia cases; meal timing not assessed.

TRE = time-restricted eating; TRF = time-restricted feeding; AD = Alzheimer’s disease; AQP4 = aquaporin-4; A*β* = amyloid-beta; NE = norepinephrine; CSF = cerebrospinal fluid; NREM = non-rapid eyemovement; FFAR3 = free fatty acid receptor 3.

**Table 3:** Proposed assessment schedule (months from randomization).

Assessment	0	3	6	9	12
PACC-5 and MoCA (primary/secondary cognitive outcomes)	•		•		•
Plasma p-tau217, p-tau181, NfL	•	•	•		•
DTI-ALPS with free-water and white-matter metrics; FLAIR/T2 for PVS and white-matter hyperintensities	•		•		•
Diurnal IL-6, TNF-α; salivary cortisol awakening response	•		•		•
Fasting insulin, glucose, HbA1c, HOMA-IR; faecal short-chain fatty acids	•		•		•
Wrist actigraphy (≥7 days), PSQI, dietary recalls, adherence review	•	•	•	•	•
DXA body composition	•		•		•
Handgrip strength, SPPB, body weight ^1^	•	•	•	•	•

• indicates that the assessment is performed at that time point. ^1^ Body weight is also checked every 2 weeks. PACC-5 = Preclinical Alzheimer Cognitive Composite-5; MoCA = Montreal Cognitive Assessment; NfL = neurofilament light chain; DTI-ALPS = diffusion tensor image analysis along the perivascular space; FLAIR = fluid-attenuated inversion recovery; PVS = perivascular space; HOMA-IR = homeostatic model assessment of insulin resistance; PSQI = Pittsburgh Sleep Quality Index; DXA = dual-energy X-ray absorptiometry; SPPB = Short Physical Performance Battery.

**Table 4:** Validity criteria for scientific hypotheses and their application to the present proposal.

Criterion	Application to This Hypothesis
Grounding in published evidence	Circadian regulation of glymphatic clearance in mice [16]; circadian disruption and AD pathology [20]; metabolic benefits of TRF in mice [9] and of early TRE in humans [14].
Testability by relevant designs	Three-arm RCT (eTRE vs. lTRE vs. habitual eating); re-analysis of longitudinal cohorts with habitual meal-timing data.
Mechanistic specificity	Hypothesized Dual-Gating model (autonomic gate: pre-sleep NE; metabolic gate: IDE availability), each step referenced and graded (Table 1); AQP4 polarization; BMAL1/CLOCK regulation.
Empirically distinguishable predictions	eTRE and lTRE should differ in DTI-ALPS and p-tau trajectories despite equal fasting duration; any cognitive benefit should be statistically mediated by glymphatic or inflammatory change (SEM criterion); a benefit without such change would favour nutrient-sensing or microbiota pathways.
Ethical testability	TRE is generally well tolerated [51,52], including in a pilot study of older adults [72]; sarcopenia and frailty risks addressed through exclusion criteria, protein targets, lean-mass monitoring, and stopping rules (Section 5.2).
Balance between novelty and established paradigms	Extends the modifiable-risk-factor model for dementia without contradicting it; builds on prior work on TRF [9] and TRE and cognition [10] by focusing on circadian timing and by proposing a testable clearance mechanism.

Criteria adapted from Misra et al. [70] and Bains [71]. TRE/TRF = time-restricted eating/feeding; eTRE/lTRE = early/late TRE; RCT = randomized controlled trial; NE = norepinephrine; IDE = insulin-degrading enzyme; AQP4 = aquaporin-4; SEM = structural equation modelling.

## Data Availability

The original contributions presented in this study are included in the article and its Supplementary Material. The full output of the AI co-scientist analysis (summary report, knowledge base, and ranked idea reports) and the code for the sample-size and mediation power simulations are available from the corresponding author upon reasonable request.

## Supplementary Materials

The following supporting information can be downloaded at https://www.mdpi.com/article/10.3390/neurosci7050106/s1, File S1: Run specification of the AI co-scientist analysis (Google Hypothesis Generation, 17 June 2026) and description of how its output was used and verified.

## Abbreviations

The following abbreviations are used in this manuscript:

AD	Alzheimer’s disease
AMPK	AMP-activated protein kinase
AQP4	Aquaporin-4
Aβ	Amyloid-beta
CSF	Cerebrospinal fluid
DAG	Directed acyclic graph
DTI-ALPS	Diffusion tensor image analysis along the perivascular space
DXA	Dual-energy X-ray absorptiometry
eTRE	Early time-restricted eating
FFAR3	Free fatty acid receptor 3
IDE	Insulin-degrading enzyme
IGF-1	Insulin-like growth factor-1
IL-6	Interleukin-6
ISF	Interstitial fluid
lTRE	Late time-restricted eating
MEQ	Morningness–Eveningness Questionnaire
MoCA	Montreal Cognitive Assessment
mTOR	Mechanistic target of rapamycin
NE	Norepinephrine
NfL	Neurofilament light chain
NREM	Non-rapid eye movement
PACC-5	Preclinical Alzheimer Cognitive Composite-5
p-tau	Phosphorylated tau
RCT	Randomized controlled trial
SCN	Suprachiasmatic nucleus
SEM	Structural equation modelling
SPPB	Short Physical Performance Battery
TNF-α	Tumour necrosis factor-alpha
TRE	Time-restricted eating
TRF	Time-restricted feeding
