# Supplementary material for: Early Time-Restricted Eating, Circadian Alignment, and Cognitive Preservation in Older Adults: A Neurobiological Hypothesis
Source: NeuroSci. 2026 Sep 30;7(5):106. doi: 10.3390/neurosci7050106 (PMC13708455; doi:10.3390/neurosci7050106)
Supplement: Supplementary file 1 [file neurosci-07-00106-s001.zip › neurosci-4559452-supplementary.pdf]

Supplementary File S1

Run specification of the AI co-scientist analysis (Google Hypothesis Generation, 17 June 2026) and description of how its output was used and verified

Manuscript: Early Time-Restricted Eating, Circadian Alignment, and Cognitive Preservation in Older Adults: A Neurobiological Hypothesis (NeuroSci, manuscript ID neurosci-4559452)

S1.1. System and date

- **System:** Google Hypothesis Generation (AI co-scientist, Google Labs), a multi-agent system that generates, critiques, ranks (tournament), and evolves research hypotheses (Gottweis et al., *Nature* 2026, 655, 487–496).
- **Date of run:** 17 June 2026.
- **Input (exact prompt):** the authors' prompt, written from the human-authored thesis draft, is recorded by the system as the **Research Summary** of the run specification (Section S1.3). At the goal-specification stage, the system structured the prompt into the *Focus Areas* and *Preferences* shown in S1.3, which the authors accepted before the run started.
- **Enabled data sources and tools:** PubMed, arXiv, and bioRxiv (data stores); UniProt, Open Targets, and ChEMBL (tools).
- **Outputs:** a run specification, a knowledge base, a summary report, and several dozen ranked idea reports. All outputs are available from the corresponding author upon reasonable request.

The run specification uses the definition of eTRE current at the time of the run (≥80% of calories before 19:00). In the manuscript, this definition was harmonized with the trial arm (≥80% of energy before 16:00; Section 3.1). The specification is reproduced unchanged.

S1.2. How the output was used and verified

Element in the manuscript	Origin	Verification by the authors
Operational definition of early eating; three mechanistic pathways; directed acyclic graph; cognitive batteries, plasma p-tau, and DTI-ALPS as outcomes	Human-authored thesis draft	Literature cited in the manuscript
Requirement that any cognitive benefit be at least partly mediated by glymphatic or inflammatory change; target population, eTRE definition, and primary outcomes	Authors' prompt (Research Summary, S1.3)	Rationale and literature in Sections 3 and 5 of the manuscript

Element in the manuscript	Origin	Verification by the authors
Three-arm design (eTRE 08:00–16:00 vs. lTRE 12:00–20:00 vs. habitual eating)	Introduced by the system at the goal-specification stage (Preferences, S1.3); the thesis draft had a two-arm design	Design rationale in Section 5 of the manuscript
“Dual-Gating” model (autonomic and metabolic gates)	AI output (several of the top-ranked research directions in the summary report)	Each step checked against independently identified primary literature; labelled as hypothesized (Table 1 of the manuscript)
Feasibility pilot, standardized timing of biological sampling, and structural equation modeling for the mediation analysis	AI output	Checked against methodological literature; adapted by the authors
Other AI-proposed mechanisms (e.g., insulin–MMP-9–dystrophin complex axis; β-hydroxybutyrate–HDAC3 epigenetic axis)	AI output	**Not adopted**: not every step could be traced to primary evidence
All bibliographic references	Authors	Each checked manually against the publisher’s record or PubMed

S1.3. Run specification (verbatim, as exported by the system)

eTRE and Glymphatic-Mediated Cognitive Resilience

Run specification · Generated on June 17, 2026 by Google Hypothesis Generation. AI can be inaccurate; double check it.

Research Summary The goal is to determine if early time-restricted eating (eTRE) serves as an independent, modifiable strategy for preventing cognitive decline in older adults by modulating circadian rhythms and specific neurobiological pathways. The research will specifically target glymphatic clearance dysfunction (AQP4-mediated clearance of amyloid-β and tau) as the primary neurobiological mechanism, with neuroinflammation (IL-6, TNF-α) and synaptic plasticity via circadian clock gene regulation (BMAL1, CLOCK) as secondary pathways.

The scope is restricted to eTRE, defined as consuming ≥80% of daily calories before 19:00 within an 8–10 hour feeding window, and maintaining a ≥3-hour gap before sleep. Success is quantified by a ≥1–2 point difference in MoCA scores over 12 months and slower decline in episodic memory and executive function compared to chronodisruptive patterns. Biological validation requires lower plasma p-tau217/p-tau181 trajectories, improved DTI-ALPS index scores, and reduced inflammatory markers. A key requirement for the hypothesis is that the association between eTRE and cognitive preservation must be partially mediated by these glymphatic or inflammatory improvements; if cognition improves without these mechanistic changes, the central model is considered weakened.

Focus Areas

Circadian-Glymphatic Synchronization and Proteostasis Investigates whether eTRE enhances glymphatic efficiency by aligning fasting and sleep with peak AQP4-dependent clearance of amyloid-β and tau. This area focuses on AQP4 polarization in astrocytic endfeet, cerebrospinal fluid dynamics, and the use of DTI-ALPS to quantify the interaction between fasting duration and sleep architecture.

Circadian Robustness as the Integrative Master Mediator Evaluates the hypothesis that the primary effect of eTRE is increasing overall circadian amplitude—specifically cortisol and melatonin rhythms—which then drives downstream cognitive benefits. This involves examining peripheral clock synchronization and rest-activity rhythm fragmentation as mediators for improved glymphatic, inflammatory, and plasticity outcomes.

Metabolic-Circadian Reprogramming and Synaptic Resilience Explores how fasting-induced metabolic signaling (via SIRT1, AMPK, and ketones like β-hydroxybutyrate) reprograms circadian and epigenetic networks to support hippocampal plasticity. This research examines the mechanistic bridge between nutrient-sensing pathways and the maintenance of episodic memory and executive function during aging.

Neuro-Immune Rhythmicity and Neurovascular Integrity Examines the restoration of daily immune oscillations (IL-6, TNF-α) and microglial clock function as a strategy to reduce chronic low-grade neuroinflammation. This area focuses on how eTRE-induced rhythmic metabolic signaling preserves the neurovascular unit and blood-brain barrier integrity.

Preferences

- Prioritize a multi-arm Randomized Controlled Trial (RCT) as the primary methodological anchor, with longitudinal cohorts as supporting evidence.
- Implement a three-arm study design comparing eTRE (08:00–16:00), lTRE (12:00–20:00), and habitual prolonged eating (>13h window).
- Focus on the eTRE vs. lTRE comparison as the primary test to isolate circadian alignment from fasting duration.
- Target episodic memory and executive function as the primary cognitive outcomes.
- Utilize DTI-ALPS (glymphatic function) and plasma p-tau217/p-tau181 as primary biological validation metrics.
- Assess circadian amplitude markers, specifically salivary cortisol rhythms, to evaluate circadian robustness as a mediator.
- Measure IL-6 and TNF-α to quantify the restoration of neuro-immune rhythmicity.
- Control for essential covariates including sleep quality, chronotype, physical activity, protein intake, and medication use.

Tools and Data Stores

- uniprot *(Tool)*
- open_targets *(Tool)*
- chembl *(Tool)*
- PubMed *(Data Store)*
- arXiv *(Data Store)*
- bioRxiv *(Data Store)*
